# The role of lymphocytes in neonatal encephalopathy

**DOI:** 10.1016/j.bbih.2021.100380

**Published:** 2021-10-20

**Authors:** Ashanty M. Melo, Nawal AB. Taher, Derek G. Doherty, Eleanor J. Molloy

**Affiliations:** a Discipline of Paediatrics and Immunology Trinity College Dublin, Crumlin, Dublin, Ireland; b Discipline of Immunology Trinity College Dublin, Crumlin, Dublin, Ireland; c Discipline of Trinity Translational Medicine Institute (TTMI), Trinity College Dublin, Crumlin, Dublin, Ireland; d Discipline of Trinity Research in Childhood Centre, Trinity College Dublin, Crumlin, Dublin, Ireland; e Discipline of Paediatrics, Children's Hospital Ireland (CHI) at Tallaght & Crumlin, Crumlin, Dublin, Ireland; f Discipline of Coombe Women and Infants University Hospital, Crumlin, Dublin, Ireland; g Discipline of Neonatology & National Children's Research Centre, Crumlin, Dublin, Ireland; h Discipline of National Children's Research Centre, Crumlin, Dublin, Ireland

**Keywords:** Neonatal encephalopathy, Hypoxic-ischaemia, Immune response, Lymphocytes, Neonatal encephalopathy, NE, central nervous system, CNS, Hypoxia-ischaemia, HI, Hypoxia-ischaemia encephalopathy, HIE, Therapeutic hypothermia, TH, natural killer, NK cells, major histocompatibility complex, MHC, White Matter Injury, WMI, Blood-brain barrier, BBB, T cell receptors, TCRs, T helper, Th, Regulatory T cells, Tregs, interleukin, IL, tumour necrosis factor-alpha, TNF-α, activating transcription factor-6, ATF6, granulocyte-macrophage colony-stimulating factor, GM-CSF

## Abstract

Neonatal encephalopathy is a syndrome characterised by abnormal neurological function often caused by a hypoxic insult during childbirth. Triggers such as hypoxia-ischaemia result in the release of cytokines and chemokines inducing the infiltration of neutrophils, natural killer cells, B cells, T cells and innate T cells into the brain. However, the role of these cells in the development of the brain injury is poorly understood. We review the mechanisms by which lymphocytes contribute to brain damage in NE. NK, T and innate T cells release proinflammatory cytokines contributing to the neurodegeneration in the secondary and tertiary phase of injury, whereas B cells and regulatory T cells produce IL-10 protecting the brain in NE. Targeting lymphocytes may have therapeutic potential in the treatment of NE in terms of management of inflammation and brain damage, particularly in the tertiary or persistent phases.

## 1 Introduction

Neonatal encephalopathy (NE) is characterised by the abnormal function of the central nervous system (CNS) developing prenatally, at birth or immediately post-delivery (Volpe, 2012). The estimated incidence of NE is 1–8 per 1000 live births worldwide (Lee and Glass, 2021). Hypoxia-ischaemia (HI) is the most widely known aetiology, but it is not solely responsible for the all cases of NE. Perinatal infections, placental abnormalities, metabolic disorders, coagulopathies and neonatal vascular stroke are also implicated in the aetiology of NE (Aslam et al., 2019). Therapeutic hypothermia (TH) is the only treatment available with an optimal response if initiated in the first 6 ​h of life, yet some infants have persistent injury (Jacobs et al., 2013).

Neuroinflammation is important for central nervous system recovery and along with systemic inflammation plays an important role in the outcome of perinatal asphyxia (Bajnok et al., 2017). The progression of brain damage is divided into three phases: the acute or primary phase happens within the first minutes after the insult, and it is defined by the primary energy failure. This energy failure is characterised by a decrease in cerebral blood flow, oxygen and glucose and a further decrease in ATP production and increase in anaerobic metabolism and lactate production. Glutamate and reactive oxygen species are released as a response to the energy failure inducing cell death (Xiong et al., 2018). The secondary phase occurs hours to days after the insult and it is characterised by the activation of microglia and astrocytes most likely via the transcription factor STAT3 and signalling molecule JAK2. STAT3 inhibition was shown to reduce microglia activation, cell death and tissue loss, as well as the recruitment of peripheral immune cells, especially leukocytes (Moynagh, 2005; Hristova, 2017). Microglia produce anti-inflammatory mediators, act as phagocytic cells and promote neurological recovery, but at the same time can produce excessive proinflammatory mediators exacerbating brain damage, hindering brain repair and neurological functional recovery (Xiong et al., 2018; Li et al., 2017).

Multiple transcription factors regulate essential cellular mechanisms and are linked to cell survival in perinatal asphyxia. Hypoxia-inducible factor (HIF) is a transcription factor sensitive to oxygen which plays an important role in cellular hypoxia. HIF-1α accumulation and binding to hypoxic response element (HRE), results in the activation of genes involve in angiogenesis, iron metabolism, and glucose metabolism by the increase of erythropoietin (EPO) and vascular endothelial growth factor (VEGF)(Bustelo et al., 2020; Jeon et al., 2019). Another important mediator is granulocyte-colony stimulating factor (G-CSF), a cytokine which is implicated in cell survival and proliferation of neutrophils via inhibition of apoptosis and inflammation (Dumbuya et al., 2021). Chemokines are small signalling proteins that induce chemotaxis of other cells. After brain injury, macrophages, astroglia, microglia and mast cells release chemokines such as CCL2, CCL3, CCL4, CCL5, CXCL1 and CXCL10 inducing the disruption of the blood-brain barrier (BBB) (Table 1; 13).Microglia and astrocytes also produce proinflammatory cytokines such as interleukin-1β (IL-1β) and tumour necrosis factor-alpha (TNF-α) (Bilbo and Schwarz, 2009), resulting in the recruitment of white blood cells to the brain (Moynagh, 2005). High levels of TNF-α and IL-1β result in the migration of neutrophils into the central nervous system (CNS) and the further disruption of the BBB (Moynagh, 2005; Dumbuya et al., 2020). TNF-α on the brain endothelium leads to the interruption of blood perfusion to the brain resulting in the exacerbation of post-ischaemic brain injury (Schmitz and Chew, 2008). Apoptotic pathways are also implicated in the complexity of the neuroinflammatory cascade after hypoxic ischaemic brain damage (Trollmann and Gassmann, 2009). Activation of activating transcription factor-6 (ATF6) (Liu et al., 2015) and caspase-3 contributes to DNA fragmentation and neuronal apoptosis (King et al., 2001). In the external mitochondrial membrane activated Bax undergoes conformational changes as a result of cytochrome *c* secreted by mitochondria, inducing more DNA fragmentation (Hardwick and Soane, 2013). Lastly the tertiary phase continues for months and years resulting in a decrease in cell plasticity and increase in dead neurons (Xiong et al., 2018). This phase is manifest by persistent inflammation and epigenetic changes leading to impaired oligodendrocyte maturation, neurogenesis and axonal growth, and lymphocyte infiltration (Li et al., 2017) (see Table 2).

**Table 1:** Mediators and transcription factors involved in NE.

Mediators and transcription factors	Stage of the disease	Release	Effect
HIF-1α	Early stages	Hypoxia	Neuroprotection (Bustelo et al., 2020)
STAT3/JAK2	Early stages	Hypoxia	Neurodegeneration (Hristova, 2017)
ATF-6 and caspase-3	Early stages	Hypoxia	Neurodegeneration (Liu et al., 2015)
CCL2, CCL3, CCL4, CCL5, CXCL1 and CXCL10 CXCL12, MIP-1α and MIP-1β, CCL5, CCL21 and CCR6.	Early stages	Macrophages Astroglia Microglia Mast cells	Neurodegeneration (Hagberg et al., 2015)
GM-CSF, IL-8, IL-1β, IL-6, IL-10, TNF-α and VEGF	Early stages	Macrophages Astroglia Microglia Mast cells	Neurodefeneration (O'Hare et al., 2017)
IFN-γ, TNF- α, granzyme B, IL-6, IL-4, IL-12, IL-17	Early and later stages in the disease	NK cell T cells Vδ2 T cells	Pro-inflammatory (Taher et al., 2021)
IL-10	Later in the disease	B cells	Anti-inflammatory (Bodhankar et al., 2013)
IL-18, TGF-β	Later in the disease	NK cells T cells	Chronic inflammation (Zareen et al., 2020)

**Table 2:** Lymphocytes in brain after HI.

Cell type	Human neonates	Animal model	Persistent inflammation in mice
**NK cells**	Unknown	↑a^d^ (Fathali et al., 2013)	–
**B cells**	↑ (Nazmi et al., 2018)	↑^b^,^d^ (Nazmi et al., 2018)	7 days post-HI
**T cells**	↑ (Nazmi et al., 2018)	↑^a^^,^^b^,^d^ (Fathali et al., 2013)	3 days post-HI to 3 months
**γδ T cells**	↑ (Albertsson et al., 2018)	↑^b^^,^^c^^,^^d^ (Albertsson et al., 2018)	6 ​h post- HI to 7 days
**iNKT cells**	Unknown	↑^b+^ (Wang et al., 2016)	24–48 ​h post- HI

Unknown: No studies have reported of NK cells and iNKT cells in the brain human brain in NE.

No studies have reported the presence of MAIT cells in human or animal models after HI.

a Rats.

b Mice.

c Sheep.

d Neonates, + adults.

Circulating immune cell activation is associated with poor outcome in brain injury (O'Hare et al., 2016). Neonates that require resuscitation at delivery have increased neutrophil and monocyte CD11b and toll-like receptor (TLR)-4 expression compared to neonatal controls (O'Hare et al., 2016). Moreover, inflammatory cytokines also play a role in NE development. Increased IL-1β, IL-6 and TNF-α in maternal urinary tract infection is associated with preterm birth, neonatal infections and neonatal brain damage leading to NE (Dammann et al., 2009). Neonates with NE have elevated cytokine levels including granulocyte-macrophage colony-stimulating factor (GM-CSF), IL-8, IL-1β, IL-6, IL-10, TNF-α and vascular endothelial growth factor (VEGF), resulting in the production and activation of TNF, TRAIL, FasL, ROS and excitotoxins leading to the exacerbation of the damage by inducing apoptosis of neuronal cells, which are associated with poor developmental outcomes and mortality (Hagberg et al., 2015; O'Hare et al., 2017). The release of IL-6, IL-8 and monocyte chemoattractant protein-1 (MCP-1) is increased by TH, resulting in a second peak at 24–56 ​h, hence supporting the concept that cytokine-mediated repair occurs at later time points (Jenkins et al., 2012). Persistent inflammation in the first week of life correlates with severe grade of NE (O'Hare et al., 2016). Cytokine dysregulation of GM-CSF, IL-18, IL-2, IL-6, IL-8 and TNF-β in response to LPS persists into childhood following NE (Zareen et al., 2020).

Neutrophils are the most abundant leukocytes and are the first immune cell recruited to the infarct after HI. Elevated neutrophil numbers in NE is associated with poor neurological outcomes (Morkos et al., 2007). Neutrophil depletion using polyclonal anti-neutrophil serum and anti-Ly6G reduces neonatal brain injury following HI in mice (Palmer et al., 2004; Doycheva et al., 2014; Yao and Kuan, 2019). The majority of studies focus on the role of neutrophils and monocytes in NE development, but little is known about the role of lymphocytes. In this review we look at the role and the possible therapeutic strategies of lymphocytes in NE.

Brain damage in acute ischemic stroke is caused by the inflammatory response during ischaemic reperfusion (Jayaraj et al., 2019), whereas infants with cerebral palsy present with persistent inflammation that may worsen the brain damage (Fleiss and Gressens, 2012). This persistent inflammation is led by lymphocytes (Liu and McCullough, 2013). In murine models of HI several immune cells are activated including T cells, B cells, natural killer (NK) cells, macrophages and dendritic cells (Hedtjarn et al., 2004). Infants with NE are 25 times more likely to have higher lymphocyte counts in the first hours of life independently of the intrapartum asphyxial insult type compared with control neonates (Phelan et al., 1998; Shah et al., 2009). Neonates with NE and neonates with acute ischaemic stroke have a significant change in the absolute lymphocyte counts and neutrophil/lymphocyte ratios during the first 12 ​h of life in neonates with NE which was not observed in those with ischaemic stroke (Povroznik et al., 2018). Low lymphocyte counts and high erythrocyte counts were associated with mortality and adverse developmental outcomes in NE (Christensen et al., 2012).

In NE, astrocytes secrete a range of chemokines resulting in the recruitment of immune cells worsening the brain injury (Hedtjarn et al., 2004). Studies in neonates and adult animal models showed that CXCL12, macrophage inflammatory proteins MIP-1α and MIP-1β, CCL5, CCL21 and CCR6 are secreted by astrocytes after HI resulting in the migration and accumulation of microglia/macrophages, CD4^+^ and CD8^+^ T cells and NK cells to the infarct (Miller et al., 2005; Bona et al., 1999; Biber et al., 2001; Park et al., 2018). Bona et al. showed that activation of microglia and CD4^+^ T cells recruited by MIP-1β, MIP-1α persisted for at least 35 days after HI, suggesting that the chronic inflammatory state in the brain is mediated by these cells (Bona et al., 1999).

## 2 Natural killer cells

NK cells account for 5% of total lymphocytes in peripheral blood. NK cells have no antigen-specific receptors and are activated by their encounter with other cells lacking the major histocompatibility complex (MHC) class I molecules or by the recognition of ligands for specific receptors. Once activated NK cells destroy target cells by cell-mediated cytotoxicity (Chaplin, 2010). Neonates have higher or similar frequencies of NK cells than adults. However, neonatal NK cells are less cytotoxic and express lower levels of L-selectin and CD54 than adults resulting in an impaired capability to adhere to target cells (Lee and Lin, 2013).

Ischaemia-reperfusion promotes expression of cyclooxygenase-2 (COX-2) and IL-15 by astrocytes inducing CD8^+^ T and NK cell infiltration and effector functions in the brain of adults and newborn mice and rats (Lee et al., 2018; Fathali et al., 2013). Fathali et al. observed that inactivation of NK cells by CD161 knockdown resulted in a decrease in brain and systemic organ atrophy and neurobehavioral deficits (Fathali et al., 2013), suggesting a key role for NK cells in brain injury and multiorgan dysfunction in NE. Investigating human peripheral blood cell phenotypes, Taher and co-workers (Taher et al., 2021) found that circulating NK cell frequencies are higher in neonates with NE compared to healthy neonates and these cells displayed activated phenotypes and more readily produced IFN-γ, TNF-α and granzyme B upon stimulation *ex vivo*.

## 3 B lymphocytes

B cells are characterised by their immunoglobulin production and account for approximately 15% of peripheral blood leukocytes (Chaplin, 2010). B cells recognise antigens through their surface immunoglobulin (Ig) receptors and TLRs. After activation B cells release soluble antibodies mediating the humoral immune response through pathogen neutralization, opsonization and complement fixation (Doherty et al., 2018). B cells mediate cognitive impairment and dementia after adult stroke (Doyle et al., 2015), but the role of B cells in neonatal brain injury has not been widely studied. Neonatal B cells lack antigenic exposure, furthermore they have impaired antibody production and an incomplete surface immunoglobulin repertoire (Li et al., 2017). Our group reported that circulating B cell numbers are higher in neonates and school-age children with NE and cerebral palsy compared to age-matched control subjects (Taher et al., 2021). B cell frequencies are higher in the injured hemisphere of the brain compared to the uninjured hemisphere 7 days after HI in a mouse model of HI-induced preterm brain injury, whereas T cells are found as soon as 3 days after HI (Nazmi et al., 2018). IL-10 reduces infarct volumes in murine ischaemic stroke (Bodhankar et al., 2013), and IL-10-secreting B cells have been found in HIE (Li et al., 2017; Bodhankar et al., 2013), suggesting B cells as essential protectors of the brain after HI. Nazmi et al. found T and B cells in the periventricular white matter and meninges of post-mortem brains from infants with periventricular leukomalacia (Nazmi et al., 2018). Infiltration of lymphocytes to the brain could contribute to brain injury by the secretion of granules and cytokines and activation of microglia, neutrophils, and endothelial cells in the brain (Nazmi et al., 2018).

## 4 Conventional T cells in NE

The majority of peripheral T cells are called conventional T cells which express αβ T cell receptors (TCRs) that recognise peptide antigens presented by MHC molecules, and the co-stimulatory receptors CD40 and CD28 (Pennington et al., 2005). CD8^+^ T cells or cytotoxic T cells specifically kill target cells that express MHC class I complex presenting antigenic peptides. In contrast, CD4^+^ T cells also known as T helper (Th) recognise MHC class II presenting peptides and respond by the release of cytokines. Th1, Th2 and Th17 ​cells are lineages of CD4^+^ effector T cells. Th1 cells produce IFN-γ, IL-2 and lymphotoxin-α (LTα) and contribute to the activation of macrophages, NK cells and cytotoxic T cells. Th2 cells regulate B cells, mast cells and eosinophils by the secretion of IL-4, IL-5 and IL-13. Th17 ​cells recruit neutrophils and macrophages and are responsible for promoting inflammation and autoimmunity. Regulatory T (Treg) cells are a subpopulation of T cells that regulate the differentiation and actions of Th1, Th2 and Th17 ​cells by the secreting of TGF-β, IL-10 and IL-35 (Barr et al., 2012; Awasthi et al., 2008). It is well known that newborn babies are Th2/regulatory T cell biased with a lack of Th1-type cytokine production by CD4^+^ T cells (Marodi, 2002). CD161^+^ CD4^+^ cells develop into Th17 ​cells, these have been observed in cord blood from term infants, however the timing of development into Th17 ​cells is still unknown (Cosmi et al., 2008). Duggan et al. reported an increase in TNF-α, IL-1β, IL-6, IL-10 and memory CD45RO^+^ T cells in cord blood and suggested this as a biomarker to predict cerebral lesions soon after delivery in preterm infants (Duggan et al., 2001). We reported that circulating T cell numbers are similar in neonates with NE and in healthy neonates but the T cells from neonates with NE more readily produced inflammatory cytokines (IFN-γ, TNF-α and IL-17) and the cytotoxic mediator granzyme B, suggesting that they are primed or activated *in vivo* (Taher et al., 2021). T cell numbers were higher in patients with cerebral palsy compared to control subjects. In HI, infiltration of CD4^+^ and CD8^+^ T cells to the brain is thought to occur in the tertiary phase of injury. CD4^+^ T cells can be detected one week after HI, whereas CD8^+^ T cells are detected two weeks after HI (Winerdal et al., 2012). T cells express the activation markers CD69 and CD25 in the damaged brain hemisphere of newborn mice up to three months after HI (Winerdal et al., 2012). Recruitment and activation of CD8^+^ T cells to the brain after CNS injury is led by IL-16 through the upregulation of CD15 and MHC class II molecules (Schwab et al., 2001). IL-16 accumulates in the cells adjacent to the injury for several days after the injury reflecting its role in the secondary and tertiary phases of brain injury (Mueller et al., 2006).

The role of T cells in HI has been studied using FTY720 a sphingosine-1-phosphate receptor agonist that blocks T cell migration. Herz et al. reported that treatment with FTY720 depleted Treg, CD4^+^ and CD8^+^ T cell in the blood resulting in a reduction of infiltrated CD4^+^ T cells and Tregs in the brain of newborn mice after HI. Lack of T cells and increased neutrophils and macrophages in the brain resulted in loss of grey and white matter (Herz et al., 2018). On the contrary, Yang *at el.* reported that treatment with FTY720 depleted Th17 ​cells from blood and brain in rat pups, resulting in less proinflammatory cytokines and a preservation of white matter (Yang et al., 2014). Moreover, in neonates with NE, Th17 cytokine production is suppressed by the increase of IL-27 expression after TH (Lowe et al., 2017). Recruitment of Th17 is most likely led by the production of IL-6, TNF-α, and IL-1β by monocytes after IL-16 stimulation. High levels of IL-6 and IL-16 were associated with more severe injury and poor neurodevelopmental outcomes (Ahearne et al., 2017).

Th1/Th2 cytokine imbalance has been reported in adults with brain damage. IL-1β and IL-6 play a key role in the early development of the inflammatory response, while the sustained inflammation in neonatal asphyxia is most likely to be maintained by TNF-α (Bajnok et al., 2017). Increased pro-inflammatory cytokines have been observed in NE. A study involving 60 white matter injury (WMI) premature neonates showed a correlation between low levels of IL-4 and IL-10 and high levels of IL-2, TNF-α and NF-κB activation with the severity of the injury. This imbalance in Th1/Th2 cytokine production along with the upregulation of NF-κB have been suggested as potential biomarkers for the early diagnosis and treatment of WMI in premature neonates (Su et al., 2018). IL-6, IL-4, IL-12, and IL-17 are elevated in neonates with NE and neonatal arterial ischemic stroke (NAIS). These cytokines are still increased in NE neonates after one month (Bajnok et al., 2018).

## 5 Innate T cells

A second class of T cells termed unconventional T cells or innate T cells recognise non-peptide antigens presented by MHC-like antigen-presenting molecules, such as CD1 and MR1. Natural killer T (NKT) cells, γδ T cells and mucosal-associated invariant T (MAIT) cells are the best characterised innate T cells. These cells differ from conventional T cells in that they possess primed/effector phenotypes and are capable of rapid expansion without the need for prior antigen exposure (LaMarche et al., 2018).

### 5.1 γδ T cells

γδ T cells are the best studied innate T cells in NE and comprise up to 5% of circulating T cells. γδ T cells are characterised by the expression of heterodimeric TCRs composed of γ and δ chains and are mainly activated in an MHC-independent manner (Allison et al., 2001; Chien et al., 2014). Once activated γδ T cells kill tumour and infected cells and stimulate monocytes, neutrophils, DCs, B cells and other T cells by the rapid secretion of cytokines, chemokines, antiviral and antimicrobial factors and via contact-dependent interactions (Chien et al., 2014). Conventional αβ T-cell responses are impaired in neonates, however, γδ T cells are already functional from early development, playing an important role in early-life immunity. Neonatal γδ T cells seem to be fully mature and able to produce IFN-γ and mount an immune response (Gibbons et al., 2009).

We used flow cytometry to investigate the numbers and functions of circulating γδ T cell subsets in a large cohort of neonates with NE, school-age children with a history of NE but who were clinically stable at the time of study, and children with cerebral palsy (Taher et al., 2021). We found striking increases in the frequencies and numbers of the Vδ2 subset of γδ T cells in neonates and school-age children with NE and cerebral palsy compared to age-matched healthy donors, whereas Vδ1 T cells were depleted from children with cerebral palsy. Upon activation with specific antigen or cytokines, Vδ2 T cells from neonates with NE more readily produced IFN-γ, TNF-α and IL-17 than Vδ2 T cells from healthy neonates, indicating that these cells were primed or activated *in vivo*. Large numbers of γδ T cells have been found in the brains of an HI mouse model, a foetal sheep asphyxia model and in post-mortem preterm infants with periventricular leukomalacia (Albertsson et al., 2018). γδ T cells are present in the brain of mice as soon as 6 h and up to 7 days after HI (Nazmi et al., 2018). However, brain damage is not IL-17F nor IL-22-dependant (Albertsson et al., 2018). Zhang et al. studied the role of γδ T cells in sepsis-induced white matter injury in a T cell receptor (TCR) δ-deficient (Tcrd^−/-^) mice model. This study showed a reduction in white matter tissue volume and altered behaviour in WT and TCR α-deficient (Tcrα^−/-^) in the presence of LPS, but not in the Tcrd^−/-^ (Zhang et al., 2017). Targeting γδ T cells might be a novel therapeutic strategy in neonatal brain injury, however, more studies must be carried out to describe the mechanism by which γδ T cells induce brain damage.

### 5.2 MAIT cells

MAIT cells comprise up to 10% of circulating T cells in humans (Dusseaux et al., 2011) and are characterised by the expression of a semi-invariant T cell receptor Vα7.2-Jα33 chain, and high expression of the C-type lectin CD161 (NKR-P1A) (Porcelli et al., 1993; Treiner et al., 2005). MAIT cells recognise microbe-derived vitamin B metabolites and small organic molecules, drugs and drug metabolites presented by MR1, an MHC-Ib-related protein (Tilloy et al., 1999; Kjer-Nielsen et al., 2012; Keller et al., 2017). When activated, MAIT cells produce granzymes and cytokines such as IFN-γ, TNF-α and IL-17A (Dusseaux et al., 2011). MAIT cells have not been widely studied in neonates. Ben Youssef et al. reported frequencies of MAIT cells in neonates to be 30 times lower than adults and increase in childhood and reaches highest frequencies in adolescence. Chen et al. also reported that MAIT cell numbers increase from birth until young adulthood and then decrease during progression to old age, but MAIT cells from children produce more IFN-γ and similar amounts of TNF-α and granzyme b than MAIT cells from young adults (Ben Youssef et al., 2018; Chen et al., 2019)*.* Our group also found that MAIT cells are found in very low numbers in neonates but they expand in children, where they account for up to 10% of circulating T cells. MAIT cells were found at normal frequencies in school-age children with NE but at reduced frequencies in children with cerebral palsy (Taher et al., 2021).

Little is known about MAIT cells and brain damage. In multiple sclerosis (MS), MAIT cells are recruited to the CNS by IL-18 (Willing et al., 2014), but MAIT functions are impaired showing decreased IFN-γ and TNF-α production (Sugimoto et al., 2016). In adults with encephalopathy caused by cirrhosis there was no difference in blood MAIT cells between patients and controls (Hegde et al., 2018), but more studies must be carried out to understand the role of MAIT cells in the brain.

### 5.3 NKT cells

NKT cells are characterised by the expression of TCRs and NK cell receptors (CD161/NK1.1) (Carreno et al., 2016) and contribute to the activation and regulation of other immune cells and have a role in tumour immunity, autoimmunity and infectious diseases (Matsuda et al., 2008). NKT cells are divided based on the TCR expression into type I NKT cells, also known as invariant (iNKT) cells, which express a semi-invariant TCRα-chain (Vα24Jα18 in humans and Vα14Jα18 in mice), whereas type II NKT cells have more variable TCR-α and -β chains repertoires (Tard et al., 2015; Singh et al., 2018). iNKT cells account to up to 0.1% of human peripheral blood T cells (Lynch, 2014). iNKT cells recognise glycolipid antigens presented by CD1d, the most widely studied antigen being the marine sponge glycolipid, α-galactosylceramide (α-GalCer) (Godfrey et al., 2015). iNKT cells activate and regulate other immune cells including DCs, T cells and B cells (Matsuda et al., 2008) by the rapid secretion of a diverse array of Th1, Th2, Th17 and Treg cytokines (Carreno et al., 2016). Due to the lack of phenotypic markers to define type II NKT cells, little is known about them. Type II NKT cells are thought to be more abundant than iNKT cells in humans. Type II NKT cells also recognise lipid antigens presented by CD1d. This subset of NKT cells do not recognise α-GalCer, but recognise mammalian glycolipids, such as sulfatides and lysophosphatidylcholine (Marrero et al., 2015). Similar to iNKT cells, type II NKT cells can secrete a range of cytokines modulating NK, T and B cell responses (Marrero et al., 2015). The brain has high amount of glycolipids furthermore the understanding of the role of NKT cells in brain injury is required (Hirabayashi, 2012).

NKT cells play a role several brain diseases including stroke, neurodegenerative diseases: MS, Alzheimer's disease (AD), Parkinson's disease (PD), Huntington's disease (HD), and amyotrophic lateral sclerosis (ALS) and CNS viral infection (Cui and Wan, 2019). NKT cell frequencies and cytokine profile in MS varies depending on the stage of the disease. For instance, IL-4 production is increased in relapsing-remitting patients compared to progressive MS patients and control subjects, whereas NKT cells express a proinflammatory profile in secondary progressive MS patients (Araki et al., 2003; De Biasi et al., 2016).

We have reported that iNKT cells are significantly expanded in neonates with NE, school-age children post-NE and children with cerebral palsy (Taher et al., 2021), suggesting a role for these cells in neuroinflammation in NE patients. In a ALS mouse model, the use of an analogue of α-GalCer delayed motor neuron death, and induced T cell infiltration to the spinal cord, prolonging the life span of the animals (Finkelstein et al., 2011). iNKT cells have been found to infiltrate the blood and brain of mice at 24 and 48 h after cerebral ischaemia. In the presence of α-GalCer, production of TNF-α and IFN-γ significantly increases neurological deficit scores and brain oedema (Wang et al., 2016). A study comparing CD1d-deficient mice, which are deficient in type I and type II NKT cells, and Jα18^−/−^ mice which are deficient in type I NKT cells only, showed an accentuated severity of renal injury in ischaemia-reperfusion injury (IRI) in mice deficient in type II NKT cells (Yang et al., 2011). In this study, sulfatide-induced activation of type II NKT cells protected the kidneys from IRI, via hypoxia-inducible factor (HIF)-1α and IL-10 pathways (Yang et al., 2011). These results suggest NKT cells as potential targets for treatment of ischaemic injury. Similar to the ischemic reperfusion renal injury, NKT cells have been observed in ischemic reperfusion brain injury. iNKT cells express a prolonged Th2-skewed immunity in stroke patients. CD1d^−/-^ mice are more susceptible to pulmonary infection after stroke, by the production of Th2-type cytokines, which correlates with the increase of IL-10 in patients with stroke, however activation of iNKT cells with α-GalCer promotes production of proinflammatory cytokines preventing stroke-associated infections (Wong et al., 2017).

## 6 Targeting lymphocytes in NE

Adoptive cellular therapy has gained a lot of attention in different fields. Reduction in brain infarct size and a prolonged improvement of neurological functions has been observed after treatment with adoptive transfer Treg cells within 24 ​h post ischaemia in mice (Li et al., 2013). Intraperitoneal injection of a CD28 superagonist monoclonal antibody (CD28SA) 3 or 6 ​h post ischaemia onset induced Treg cells expansion, leading to IL-10 production and the reduction of brain injury after adult mice cerebral ischaemia *in vivo* (Na et al., 2015). Another potential treatment is targeting NK and T cell infiltration. During Ischaemia-reperfusion IL-15 produced by astrocytes induces NK cell infiltration. IL-15 blockade has been shown to reduce the effector function of NK, CD8^+^ T cells and CD4^+^ T cells in brain of WT mice after ischaemia-reperfusion, resulting in the reduction of the infarct size and improvement of the motor and locomotor activity (Lee et al., 2018). Our own data (Taher et al., 2021) that suggest roles for Vδ2 T cells and iNKT cells in neuroinflammation in NE and cerebral palsy, indicate that therapeutic strategies involving these cells that are currently being tested in cancer patients, may be adapted for the treatment of NE (Lowe et al., 2017; Hegde et al., 2018). However, future research into the mechanisms by which these cells contribute to NE is required before these immunotherapies can be translated to humans with NE.

## 7 Concluding remarks

NE is characterised by the damage of the brain at birth or post-delivery (Volpe, 2012). Neuroinflammation plays an important role in perinatal asphyxia. After a hypoxic insult the decrease in cerebral blood flow, oxygen and glucose induces cell death and microglia activation (Xiong et al., 2018). Activation of microglia and astrocytes by a hypoxic insult promotes neurological recovery, however, it also leads to microglia activation, cell death and tissue loss, as well as the recruitment of peripheral immune cells, especially leukocytes (Moynagh, 2005; Hristova, 2017). Early stages of the damage is led by the release of chemokines such as CCL2, CCL3, CCL4, CCL5, CXCL1 and CXCL10 by macrophages, astroglia, microglia and mast cells leading to the disruption of the BBB and production of IL-1β and TNF-α (Bilbo and Schwarz, 2009), resulting in the recruitment of white blood cells to the brain (Moynagh, 2005).

Lymphocytes play an important role in the protection and degeneration of the brain after a hypoxic insult. Circulating immune cell activation is associated with poor outcome in brain injury and are the main cause of persistent inflammation (O'Hare et al., 2016). In NE, astrocytes and microglia release several cytokines such as IL-15, IL-8 and IL-16 and chemokines such as CXCL12, MIP-1β, MIP-1α, CCL5, CCL21 and CCR6 inducing NK cells, B cells, T cells and innate T cells to migrate to the infarct (Bilbo and Schwarz, 2009). Migration and accumulation of lymphocytes is associated with a chronic response resulting in the exacerbation of the brain damage days and even months after the hypoxic insult by the production of proinflammatory cytokines like IFN-γ, TNF-α and IL-17 (Mueller et al., 2006).

**Fig. 1:**
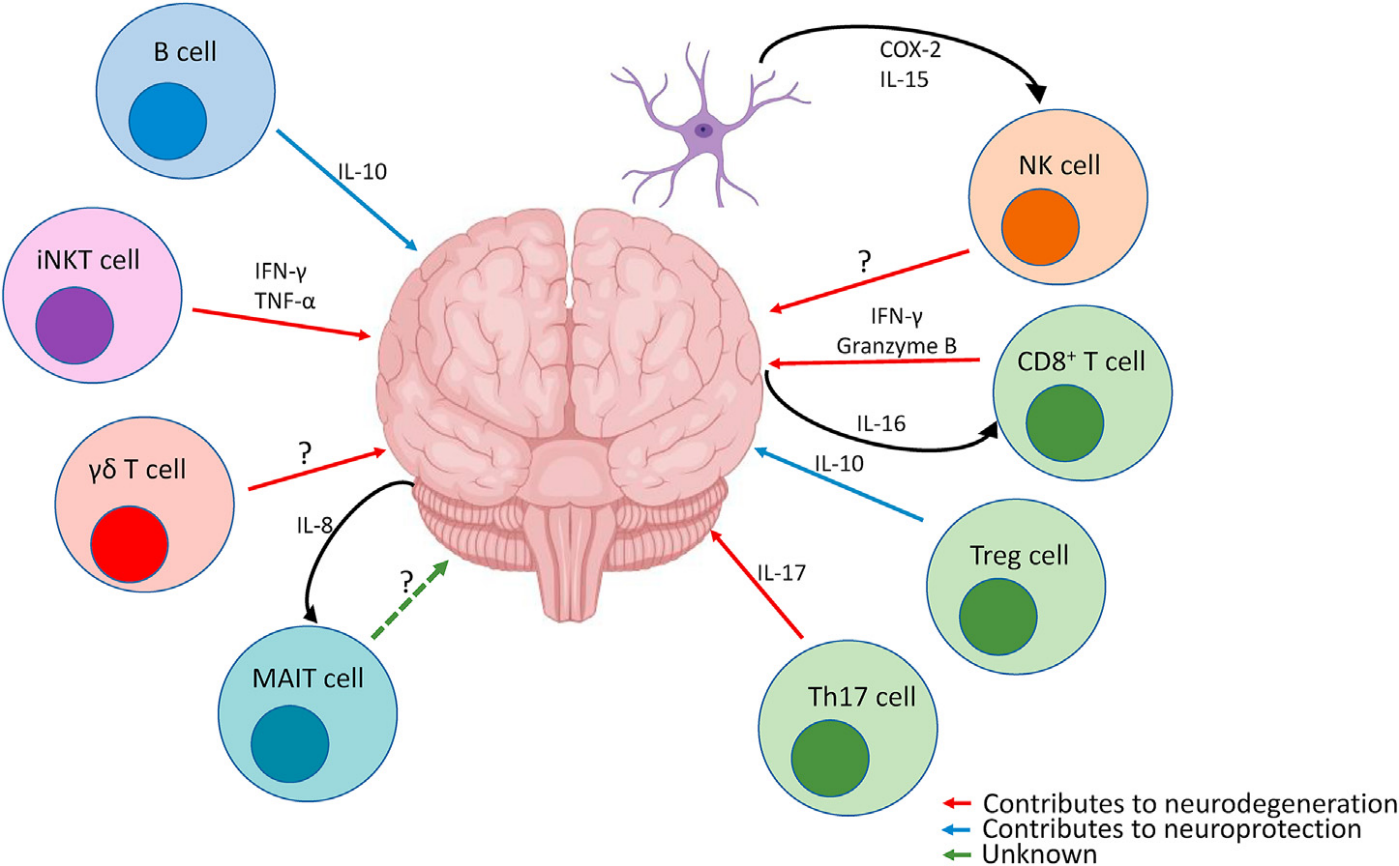
**Lymphocytes role in NE.** After HI astrocytes release several cytokines and chemokines attracting immune cells into the infarct area. CD8^+^ T cells and NKT cells contribute with brain damage by the production of IFN-γ, TNF-α and granzyme B whereas Th17 ​cells contribute to the damage by the production of IL-17. γδ T cells and NK cells play a role in neurodegeneration, however, no specific mechanism of action has been described. On the contrary, B cells and Tregs protect the brain by the production of IL-10. Up to this day, the role of MAIT cells has not been described in NE. (Red arrows represent neurodegeneration, blue arrows represent neuroprotection, green arrows represent unknown function). (For interpretation of the references to colour in this figure legend, the reader is referred to the Web version of this article.)

Therapeutic hypothermia (TH) is the only treatment available for NE, however timing is very important and the response is not optimal as some infants present persistent injury (Jacobs et al., 2013). The study of the lymphocytes in NE has open a new field of potential therapies to improve the outcome in NE. Treatment with IL-15 a cytokine known to induce infiltration of NK, CD8^+^ T cells and CD4^+^ T cells could potential reduce the inflammation and further damage of the brain in NE (Lee et al., 2018). Targeting NK and T cells after hypoxic-ischaemia have shown promising results to treat NE, targeting lymphocytes using therapeutic strategies for cancer may be useful in NE. Finding new ways to target lymphocytes might offer a new treatment potential to persistent inflammation in NE and the further worsening of the brain damage especially in the tertiary phase(see Fig. 1).

## Declaration of competing interest

None.
